# Small Phytoplankton Shapes Colored Dissolved Organic Matter Dynamics in the North Atlantic Subtropical Gyre

**DOI:** 10.1029/2019GL084699

**Published:** 2019-11-08

**Authors:** Emanuele Organelli, Hervé Claustre

**Affiliations:** ^1^ Université, CNRS, Laboratoire d'Océanographie de Villefranche, LOV, F‐06230 Villefranche‐sur‐Mer France

**Keywords:** Coloured dissolved organic matter, pico‐phytoplankton, subtropical gyres, carbon cycle, bio‐optics, BGC‐Argo floats

## Abstract

The North Atlantic subtropical gyre (NASTG) is a model of the future ocean under climate change. Ocean warming signals are hidden within the blue color of these clear waters and can be tracked by understanding the dynamics among phytoplankton chlorophyll ([Chl]) and colored dissolved organic matter (CDOM). In NASTG, [Chl] and CDOM are strongly correlated. Yet, this unusual correlation for open oceans remains unexplained. Here, we test main hypotheses by analyzing high spatiotemporal resolution data collected by Biogeochemical‐Argo floats between 2012 and 2018. The direct production of CDOM via phytoplankton metabolism is the main occurring mechanism. More importantly, CDOM dynamics strongly depend on the abundance of picophytoplankton. Our findings thus highlight the critical role of these small organisms under the ocean warming scenario. Picophytoplankton will enhance the production of colored dissolved compounds and, ultimately, impact on the ocean carbon cycle.

## Key Points

- Colored dissolved organic matter is produced via phytoplankton metabolism in the North Atlantic subtropical gyre
- Picophytoplankton shapes the dynamics of colored dissolved organic matter within the mixed layer of the North Atlantic subtropical gyre
- Picophytoplankton may become a critical source of colored dissolved organic matter under ocean warming

## 1 Introduction

Among the clearest world oceans, the North Atlantic subtropical gyre (NASTG; 14–30°N; Longhurst, 2007) is less blue than the other oligotrophic subtropical zones. NASTG is characterized, at its surface, by a higher content in colored dissolved organic matter (CDOM) that enhances light absorption in the ultraviolet (UV) and blue regions of the electromagnetic spectrum (Morel et al., 2010). CDOM thus attenuates the penetration of UV and blue light with depth and modifies the color of the ocean (Morel & Prieur, 1977). Modifications in CDOM light absorption track climate change signals (Dutkiewicz et al., 2019), and ultimately may affect the ocean's heat budget (Kim et al., 2018). Because of ocean warming due to climate change, NASTG is expanding and thus represents how the future ocean will look like (Polovina et al., 2008). Yet, the fate of CDOM in this region will remain unpredictable until main drivers shaping its dynamics are unraveled.

In the upper NASTG, the dynamics of space‐derived CDOM light absorption coefficients and phytoplankton chlorophyll concentrations are tightly correlated with minima in summer and maxima in winter (Morel et al., 2010). Such a synchronous correlation was unexpected in open‐ocean waters (Nelson & Siegel, 2013) and for the close Sargasso Sea (Hu et al., 2006; Nelson et al., 1998). Indeed, in open‐ocean waters, a delay between phytoplankton chlorophyll and CDOM maxima is typically observed (Nelson & Siegel, 2013). This delay is generally associated with CDOM production through phytoplankton digestion as operated by heterotrophic bacteria (Nelson et al., 1998) and excludes active CDOM release by phytoplankton as observed in laboratory (Romera‐Castillo et al., 2010; Seritti et al., 1994). Hence, to explain the tight correlation observed between CDOM and chlorophyll, Morel and coauthors (2010) formulated three hypotheses: (i) CDOM is directly released into seawater via phytoplankton metabolism and it is not a by‐product of heterotrophic bacteria; (ii) lower solar radiation reduces CDOM photodegradation in winter and enhances phytoplankton chlorophyll concentrations; (iii) winter convection replenishes the upper layer with CDOM and nutrients from deepest reservoirs, while nutrients favor in turn phytoplankton growth. A combination of all the three hypotheses was ultimately considered as the most likely scenario. However, because of the lack of in situ vertical profiles of other bio‐optical, physical, and biogeochemical variables, no hypothesis was confirmed, and main drivers of CDOM in NASTG are still unexplained.

Four Biogeochemical‐Argo (BGC‐Argo) floats have routinely acquired vertical profiles of radiometry, phytoplankton chlorophyll fluorescence (FChl), and hydrology in NASTG for 6 years (2012–2018). Here, we analyze the relationship between proxies of CDOM light absorption coefficients and chlorophyll concentrations within the mixed layer, and explain what are the main drivers of the observed temporal dynamics. We then propose a possible scenario for CDOM in expanding clear waters, such as NASTG, under increasing ocean warming.

## 2 Materials and Methods

**Figure 1:**
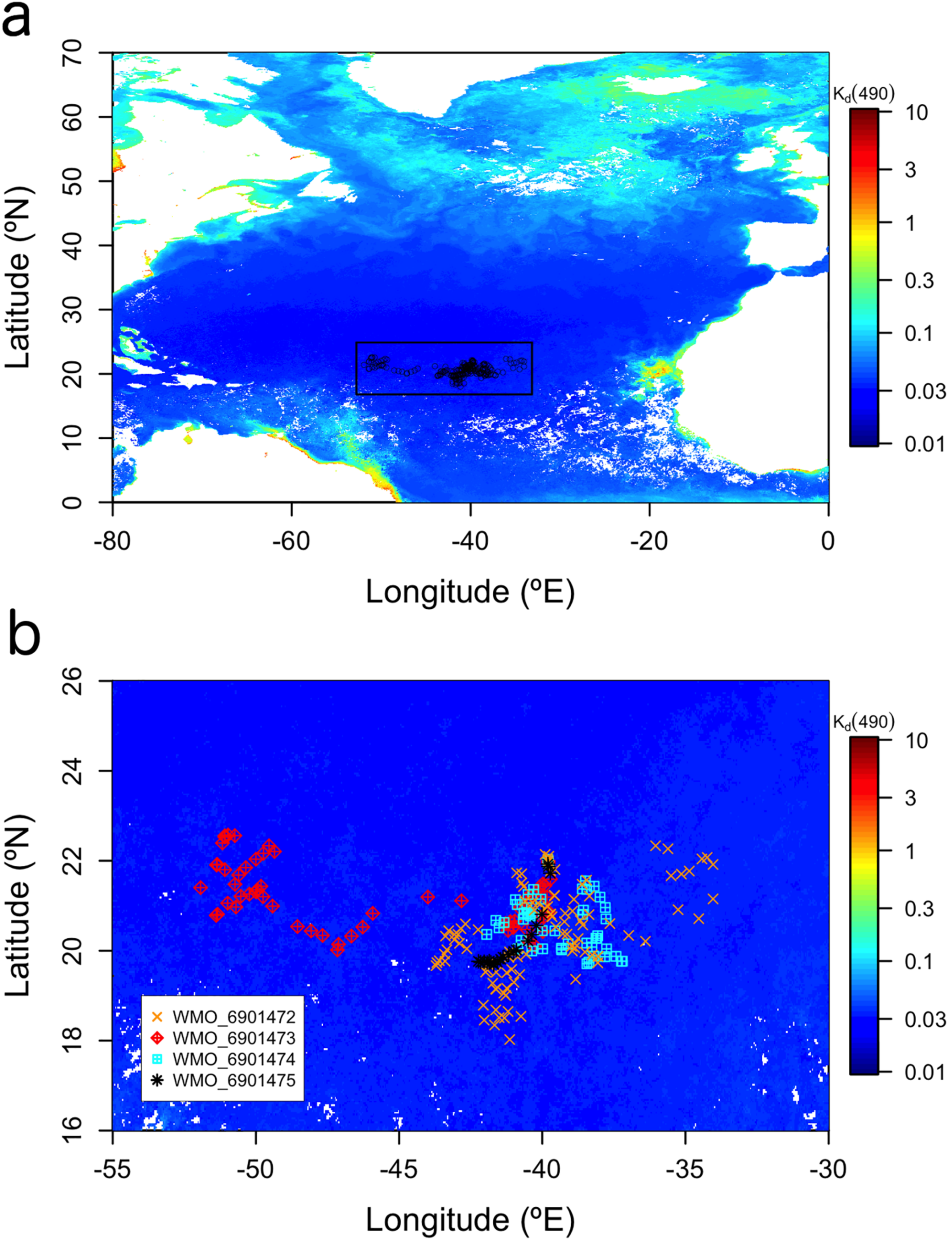
The North Atlantic subtropical gyre. (a) Area (black rectangle) and stations (black open circles) sampled by four Biogeochemical‐Argo floats between 2012 and 2018; (b) Float trajectories. In both panels, only stations after quality control of variables are shown (*n* = 235). The WMO numbers of each BGC‐Argo float (and the dates of the first and last quality‐controlled profiles) are 6901472 (24/10/2012–19/09/2018), 6901473 (25/10/2012–04/07/2016), 6901474 (24/10/2012–15/06/2015), and 6901475 (26/10/2012–24/01/2013). Sampled stations are superimposed onto the July 2014 Ocean Colour European Space Agency Climate Change Initiative (v3.1) monthly composite of the diffuse attenuation coefficient for downward irradiance at 490 nm (*K*_d_(490); units of per meter). Low *K*_d_(490) coefficients are associated with very clear waters. Float trajectories before quality control are shown in Figure S1. WMO = World Meteorological Organization.

Four PROVOR CTS‐4 profiling floats were deployed in NASTG and operated between 2012 and 2018 (Figure 1; see supporting information, Figure S1). PROVOR CTS‐4 floats were configured and deployed according to standard procedures (Bittig et al., 2019). Each float collected 0–1000 m vertical profiles of temperature (T), salinity (S), dissolved oxygen concentration, FChl, and fluorescent dissolved organic matter (FDOM). BGC‐Argo floats also acquired 0–250 m vertical profiles of downward irradiance at 380 and 490 nm (*E*_*d*_(380) and *E*_*d*_(490), respectively), and instantaneous Photosynthetically Available Radiation (PAR) integrated between 400 and 700 nm. Vertical profiles were acquired every 1 up to 10 days under high solar elevation angles (>30°; i.e., around local noon). Either raw or quality‐controlled data were gathered from Coriolis Global Data Assembly Centre (ftp://ftp.ifremer.fr/ifremer/argo/dac/coriolis/; Argo, 2019). All these data were already checked for sensor malfunctioning and bio‐fouling issues (Argo Data Management Team, 2017). Each variable was quality‐controlled as described below.

Pressure, T and S were collected by a SBE‐41 CP conductivity‐T‐depth sensor (Sea‐Bird Scientific). Data were quality‐controlled according to standard, internationally‐accepted protocols as discussed by the Argo Data Management Team (Wong et al., 2019). T and S were then used to calculate seawater potential density anomaly and, ultimately, to estimate the thickness of the mixed layer. The mixed layer depth (MLD) was defined as the depth at which seawater density was >0.03 kg m^−3^ with respect to its value at 10 m (de Boyer Montégut et al., 2004). Finally, T and S mean values within the mixed layer were computed from quality‐controlled vertical profiles.

Dissolved oxygen concentrations were acquired by an Aanderaa Data Instrument 4330 optode and quality‐controlled following Thierry et al. (2018). Quality‐controlled profiles were used, together T and S, as input of the neural network‐based method CArbonate system and Nutrients concentration from hYdrologycal properties and Oxygen using a Neural‐network (Bittig et al., 2018; Sauzède et al., 2017) to obtain high‐quality vertical profiles of nitrate concentrations. Nitrate vertical profiles were then used to estimate the depth of the nitracline (*Z*_NO__3_), that is, the depth which separates upper nitrate‐depleted from deeper nitrate‐rich waters. *Z*_NO__3_ was defined as the depth at which the isocline 1 μM was located (Pasqueron de Fommervault et al., 2015).

FChl vertical profiles were acquired by a Sea‐Bird WetLABS Environmental Characterization Optics sensor with excitation at 470 nm and emission at 695 nm. FChl measurements were converted in phytoplankton chlorophyll concentration ([Chl], units of milligrams per cubic meters) and then quality‐controlled according to Schmechtig et al. (2015; 2018). Briefly, the quality control procedures consisted in applying a test range for measured values and corrections for nonphotochemical quenching (Xing et al., 2012) and overestimation due to factory calibration (Roesler et al., 2017). [Chl] values <0.0146 mg m^−3^ were removed because these values were below the sensor's detection limit. Averaged [Chl] values within the mixed layer were computed from quality‐controlled vertical profiles. Finally, mean [Chl] values were used to estimate the relative abundances of picophytoplankton (<2 μm), nanophytoplankton (2–20 μm), and microphytoplankton (>20 μm) following the method described by Hirata et al. (2011). This method also allowed to distinguish between the contributions of picocyanobacteria and picoeukaryotes within picophytoplankton as well as those of haptophytes and green algae within the nanophytoplankton size class.

FDOM vertical profiles (units of parts per billion of quinine sulfate) were acquired by a Sea‐Bird WetLABS Environmental Characterization Optics sensor with excitation at 370 nm and emission at 460 nm, and quality‐controlled following Organelli, Barbieux, et al. (2017). Values outside the range reported by the manufacturer, and negative and positive spikes were removed. Remaining outliers were additionally filtered and, finally, vertical profiles were aligned to the median value between 950 and 1,000 m of the first profile to correct for possible sensor drifts (Organelli, Barbieux, et al., 2017). Note that because of sensor issues, the temporal coverage of FDOM quality‐controlled data is shorter than for other variables, and profiles were directly downloaded from Organelli, Barbieux, et al. (2017). Average FDOM values within the mixed layer were computed from quality‐controlled vertical profiles. BGC‐Argo FDOM measurements report only on the fraction of CDOM associated with humic material (Nelson & Gauglitz, 2016; Stedmon & Nelson, 2015).

*E*_*d*_(380), *E*_*d*_(490), and PAR were collected by a Satlantic Inc. multispectral OCR‐504 radiometer and quality‐controlled following Organelli et al. (2016). This quality control accepts radiometric profiles acquired under clear or overcast sky as soon as these conditions remain stable during the cast. Hence, the quality control first removed profiles collected under unstable sky and sea conditions. Then, remaining profiles were checked to identify and remove dark measurements at depth, sporadic atmospheric clouds, and wave focusing in the upper part of the profile. For each quality‐controlled profile, *E*_*d*_(380), *E*_*d*_(490), and PAR just below the surface were computed through extrapolation within the first optical depth (*Z*_*pd*_) using a second‐degree polynomial function (Organelli et al., 2016). *Z*_*pd*_ was defined as *Z*_*eu*_/4.6, where *Z*_*eu*_ is the depth at which PAR is reduced to 1% of its value just below the surface. The average quantity of PAR within the mixed layer (PAR_ML_) was computed following Morel et al. (2010). Because all measurements were acquired around noon when solar elevation and irradiance are the highest, PAR_ML_ thus represents the maximal dose of light within the mixed layer over a 24‐h period.

To calculate a proxy of CDOM light absorption coefficients (i.e, *K*_bio_(380); Morel, Claustre, et al., 2007), *E*_*d*_(380) vertical profiles were used to compute the diffuse attenuation coefficient for downward irradiance within the mixed layer (*K*_*d*_(380); units of per meter). Quality‐controlled E_d_(380) profiles were first binned in 1‐m intervals. *K*_*d*_(380) was then derived as the slope of a linear fit between the natural logarithm of *E*_*d*_(380) and depth, and quality‐controlled following Organelli et al. (2017). The contribution of pure seawater was then subtracted from *K*_*d*_(380) to obtain *K*_bio_(380) (Morel & Maritorena, 2001). Note that this decomposition is accepted in NASTG because samples were acquired in very clear waters and under high solar elevation angles (Lee et al., 2018; Morel & Maritorena, 2001). Finally, K_bio_(380) coefficients were divided by mean [Chl] values within the mixed layer to obtain the chlorophyll‐specific attenuation coefficient for CDOM (*K*_star_(380); units of square meters per milligram of chlorophyll).

The same procedure as described above was applied to quality‐controlled *E*_*d*_(490) vertical profiles to estimate *K*_bio_(490) coefficients. Despite of being influenced by light absorption of phytoplankton accessory pigments, *K*_bio_(490) is a statistically significant proxy of [Chl] as those computed for wavelengths at which chlorophyll light absorption is the highest (e.g., 440 nm; Morel, Huot, et al., 2007).

Statistical analyses were conducted on monthly climatology. For each variable, the monthly value is the mean (± standard deviation) computed by using all data collected by the four BGC‐Argo floats. For each monthly value, the percent coefficient of variation (CV%) was computed as: 100 × (standard deviation/mean). To calculate the relationships among *K*_bio_(380) and other variables, multiple and simple linear regressions were applied. The *r*‐squared and the Pearson's correlation coefficient (*r*) were calculated, and the significance of the fits was tested by a two‐tailed Student's *t*‐test. Before applying parametric tests, we tested the dataset for normality and data were log‐normal distributed (but *Z*_*eu*_ and *PAR*_ML_), accordingly with Campbell (1995). Note that because the procedure by Organelli et al. (2016) to quality control radiometry is stricter for PAR than *E*_*d*_(380), only the stations with good PAR profiles (*n* = 235; Figure 1) were finally used to calculate climatology for all variables (but *Z*_NO__3_).

## 3 Results and Discussion

**Figure 2:**
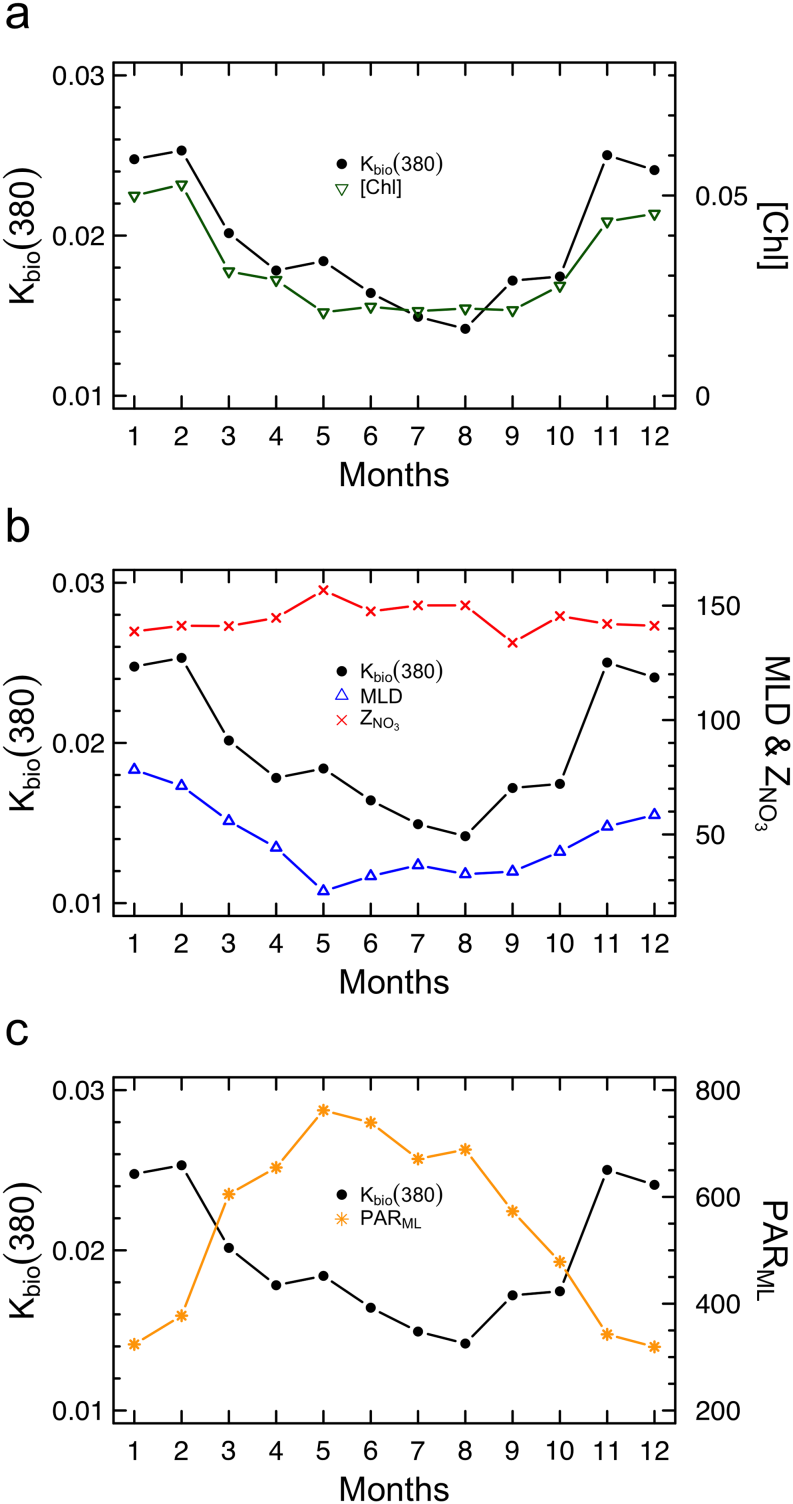
Monthly climatology of *K*_bio_(380) (units of m^−1^) and other biogeochemical and physical variables within the mixed layer. (a) K_bio_(380) and phytoplankton chlorophyll concentrations ([Chl]; units of milligrams per cubic meters); (b) K_bio_(380), the mixed layer depth (MLD; units of m) and the depth of the nitracline (Z_NO__3_; units of m); (c) K_bio_(380) and the average value of the Photosynthetically Available Radiation at solar noon within the mixed layer (PAR_ML_; units of μmol quanta m^−2^ s^−1^). The coefficients of variation (CV%) for each monthly average are listed in Table S1.

In NASTG, despite of the lowest values observed among the world oceans (Organelli, Claustre, et al., 2017), K_bio_(380) and [Chl] are characterized by a clear seasonal cycle within the mixed layer (Figure 2a; see supporting information Table S1). These cycles are synchronized, and both show minima in summer and maxima in winter accordingly with Morel et al. (2010) spaced‐based observations. *K*_bio_(380) significantly decreases in correspondence with shallower MLDs (Figure 2b; see supporting information Table S2) and low S (see supporting information Figure S2; Table S2), as well as when PAR_ML_ increases (Figure 2c; Table S2). *K*_bio_(380) also decreases in correspondence with deeper *Z*_NO__3_ (Figure 2b) but not in a significant manner (Table S2), while a cross‐correlation analysis suggests dependence of changes in T within the mixed layer on *K*_bio_(380) (Figure S2). Thus, a multiple regression analysis using [Chl], MLD, PAR_ML_, and S as independent variables revealed that these variables together explain more the 90% of the monthly variability in *K*_bio_(380) (*r*‐squared = 0.91, *p* < 0.01; Table S2). Yet, PAR_ML_, MLD, and S do not add significant contribution to shaping *K*_bio_(380) with respect to [Chl] (statistics for comparison among variables are in Table S2). [Chl] alone explains almost the totality of monthly variability in *K*_bio_(380) coefficients (*r*‐squared = 0.87, *p* < 0.01; Table S2), and thus, CDOM dynamics in the sampled area. Such results tell us that CDOM is directly produced by phytoplankton in NASTG and help rejecting the two other hypotheses previously formulated by Morel et al. (2010).

Vertical mixing does not replenish CDOM within the mixed layer in winter. CDOM light absorption coefficients measured in NASTG at the same time of the year are homogeneously distributed down to 250‐m depth (Nelson et al., 1998) and within deep euphotic layers (i.e., 140 m; Iuculano et al., 2019). Yet, the highest MLD values observed in January are of 78 ± 26 m (Figure 2b; Table S1) while the deepest MLD of 129 m has been recorded in February. MLD values and seasonality as reported by the four BGC‐Argo floats are consistent with previous observations in NASTG (de Boyer Montégut et al., 2004; Reverdin et al., 2015). This implies that the mixed layer in NASTG is not thick enough to allow CDOM upwelling from deep reservoirs.

Photodegradation yields a minor impact on CDOM dynamics in NASTG. CDOM photodegradation is expected to depend on the time the water mass is exposed to solar light (Yamashita et al., 2013). Substantial CDOM photochemical destruction thus occurs months after the highest annual radiation rates (Organelli et al., 2014). In NASTG, lowest *K*_bio_(380) coefficients were therefore expected in September. At this time, PAR_ML_ decreases while the mixed layer is still shallow as in the previous months. The same water mass has therefore been exposed to the highest light doses for more than 3 months (Figure 2c) which would have enhanced photodegradation. Moreover, photodegradation of dissolved compounds could also be inhibited by the occurring nitrate limitation that, ultimately, reduces the susceptibility of surface CDOM to illumination conditions (Swan et al., 2012). However, illumination is unlikely a limiting factor in NASTG despite of the observed seasonal variations (Figure 2c; Morel et al., 2010). For example, the depth of the euphotic zone *Z*_eu_ is similar regardless of the season (103 ± 11‐121 ± 6 m; see supporting information Figure S3; Table S1). Such a high light availability therefore suggests that the degree of CDOM photodegradation is similar during all the year in NASTG.

vThe relevance of phytoplankton metabolism as primary source of CDOM in NASTG depends on the extent to which observed [Chl] dynamics are also related to changes in carbon biomass in addition to photoacclimation. Photoacclimation has been previously shown as the main driver of [Chl] in this region and for the other oligotrophic subtropical gyres (Barbieux et al., 2018; Mignot et al., 2014). However, in NASTG, modifications in [Chl] due to changes in carbon biomass also occur, as highlighted by the observed strong correlation between [Chl] and *K*_bio_(490) (*r* = 0.84, see supporting information Figure S4). [Chl] is derived from fluorescence measurements, and thus, its conversion to carbon biomass strongly depends on phytoplankton species composition and growth, and illumination conditions (Cullen, 1982). Conversely, *K*_bio_(490) coefficients are proxies of phytoplankton pigment light absorption and thus are inherently more related to chlorophyll concentration and actual carbon biomass than fluorescence‐derived [Chl] (Roesler & Barnard, 2013). Thus in winter, for example, observed highest chlorophyll concentrations and *K*_bio_(490) values indicate an increase in carbon biomass, which is likely favored by the upwelling of nitrates into the mixed layer through diffusion from a shallower nitracline than from spring to autumn (Lewis et al., 1986; Figure 2b).

**Figure 3:**
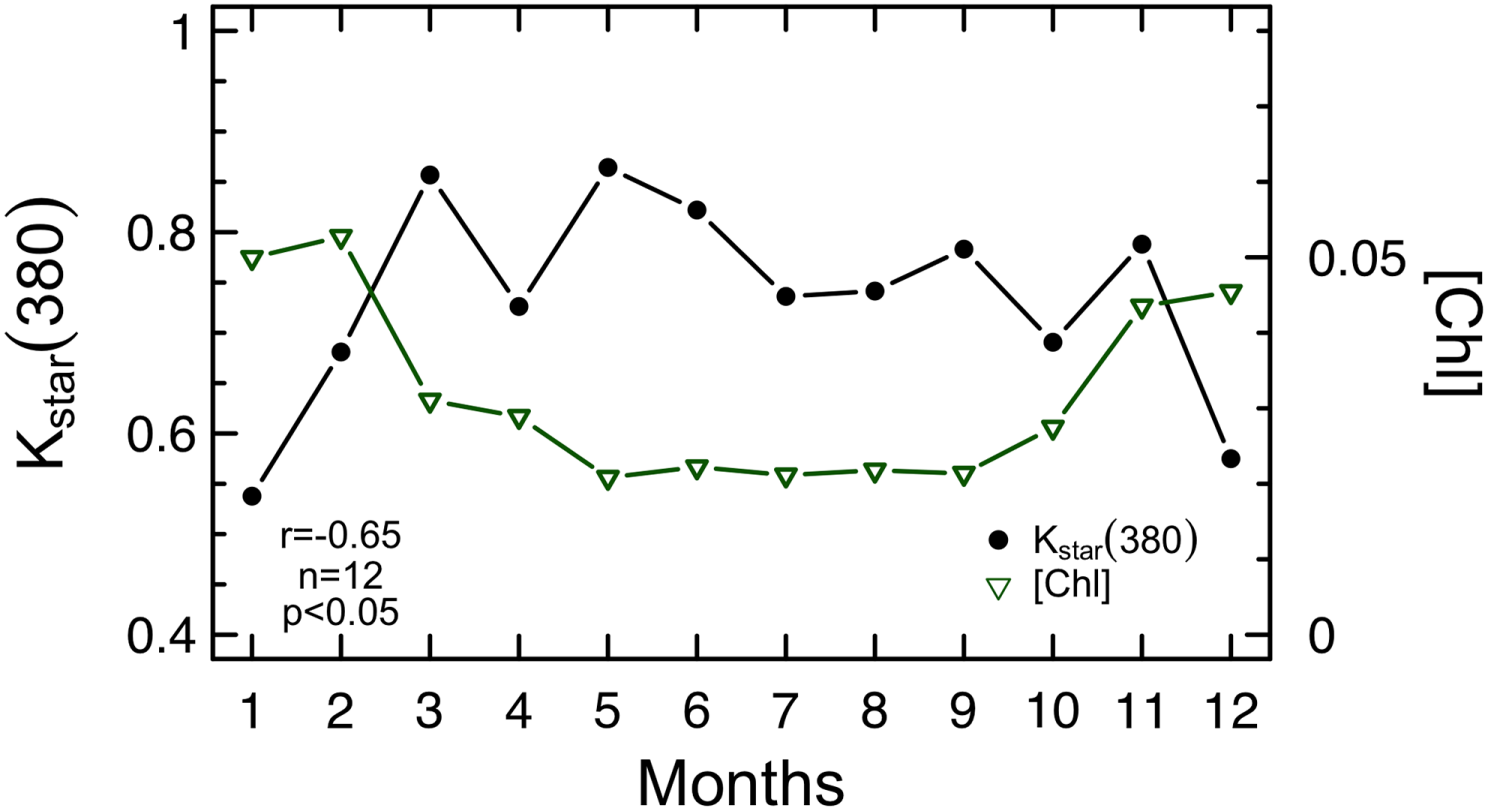
Colored dissolved organic matter light absorption per unit of [Chl] is high from spring to autumn. Monthly climatology of phytoplankton chlorophyll concentration ([Chl]; units of milligrams per cubic meters) and chlorophyll‐specific diffuse attenuation coefficient for downward irradiance at 380 nm (*K*_star_(380); units of square meters per milligram of chlorophyll) within the mixed layer. Statistics for linear correlation between *K*_star_(380) and [Chl] are shown. The coefficients of variation (CV%) for each monthly average are listed in Table S1.

From spring to autumn, when carbon biomass is low, phytoplankton releases more CDOM per unit of [Chl] than in winter (Figure 3) which supports active CDOM exudation via photosynthetic overflow (Thornton, 2014). In summertime, for example, high light conditions (Figure 2c) boost photosynthesis but nutrient limitation cannot fully support cell growth (Marañón, 2005). Photosynthesis is thus faster than phytoplankton growth and leads to an intracellular accumulation of organic compounds (Fogg, 1983). When the cellular storage capacity is overwhelmed, phytoplankton releases into the seawater any exceeding products. Such a mechanism characterizes healthy growing cells, but it may account only for about 10% of the productivity (Myklestad et al., 1989), which explains the lower *K*_bio_(380) values observed in summer than in winter (Figure 2).

**Figure 4:**
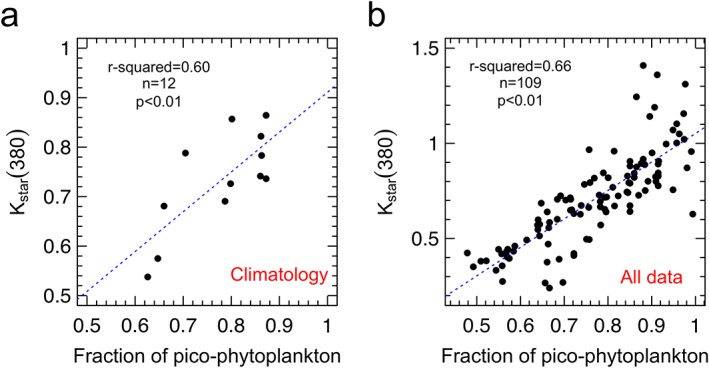
Picophytoplankton shapes CDOM dynamics. Chlorophyll‐specific diffuse attenuation coefficient for downward irradiance at 380 nm (K_star_(380); units of square meters per milligram of chlorophyll) as a function of the fraction of picophytoplankton (units of percentage): (a) monthly climatology; (b) all data. Blue dashed line is the linear fit to all data. Statistics are shown. The coefficients of variation (CV%) for each monthly average are listed in Table S1.

Picophytoplankton and especially cyanobacteria such as the genera *Prochlorococcus* and *Synechococcus* shapes *K*_star_(380) and thus CDOM light absorption temporal dynamics in NASTG (Figure 4; see supporting information Figure S5). The relevance of picophytoplankton is confirmed by growing literature in such very oligotrophic areas both for the production of dissolved (Roshan & DeVries, 2017; Teira et al., 2001) and colored (Iuculano et al., 2019; Zhao et al., 2017) organic matter. Here we show that cyanobacteria dominate all the year (Figure S5), accordingly with previous studies (e.g., Heywood et al., 2006). More importantly, cyanobacteria maxima (up to 56% on average) are observed for the highest *K*_star_(380) coefficients, that is, from spring to autumn when these organisms release more CDOM per unit of carbon biomass (Figure 3). During the same period, the contribution of picoeukaryotes also increases up to 31% (Figure S5). Among the others size classes, microphytoplankton contributes <3% during all the year while nanophytoplankton follows the opposite seasonal trend than picophytoplankton (Figure S5; Table S1). The relative contribution of nanophytoplankton, which is mainly dominated by haptophytes, is the lowest in summer (13% on average) when K_star_(380) coefficients are the highest (Figure S5; Table S1).

The increasing importance of smallest phytoplankton during the most stratified and illuminated period of the year allows, therefore, looking at picophytoplankton as a new potential source of CDOM whose role may become critical under the ocean warming scenario. Such a scenario leads to increased exposure of surface waters to light and reduced nutrient supply from the deep ocean due to enhanced stratification of the water column. Under such environmental conditions, picophytoplankton is expected to be more resilient than other groups (Agustí et al., 2019; Flombaum et al., 2013; Morán et al., 2010). In such circumstances, picophytoplankton will therefore boost active CDOM release into seawater.

It may be argued that results and discussions here presented deal on variability of the *K*_bio_(380) coefficient which is only a proxy of CDOM light absorption coefficient. Previous studies in the clearest oligotrophic world oceans have shown that CDOM dominates the light absorption budget at 380 nm (Bricaud et al., 2010) and light attenuation in the UVs (Morel, Claustre, et al., 2007; Siegel & Michaels, 1996; Smyth, 2011). Unfortunately, we do not have coincident light absorption data to prove this statement. Yet, other possible sources that affect light attenuation in the UVs, such as light absorption by mycosporine‐like amino acids and non‐algal particles (NAP), can be excluded or considered negligible. Mycosporine‐like amino acids are photoprotective pigments that have been observed in cyanobacteria as well as in a variety of nanophytoplankton and microphytoplankton species (Carreto & Carignan, 2011), but their light absorption maxima occurs between 310 and 360 nm (Carreto & Carignan, 2011). NAP light absorption at 380 nm contributes less than 20% to total non‐water absorption in clear oligotrophic waters (Bricaud et al., 2010). More importantly, NAP optical contribution does not significantly vary along the year in NASTG (Bellacicco et al., 2019) so that NAP cannot help shaping the temporal variability of *K*_bio_(380) coefficients.

Though this study highlights the main role of phytoplankton on a climatology scale, the dependence of CDOM dynamics on other processes cannot be totally excluded at a shorter scale. Heterotrophic bacteria may still act as a source of CDOM (Nelson et al., 1998), and rapid convection and mesoscale activity can bring CDOM within the mixed layer from deep reservoirs (Nelson et al., 2007). In addition, in NASTG, CDOM temporal dynamics could also depend on the varying lability of chemical compounds. For example, Zhao et al. (2017) have shown that picophytoplankton releases CDOM that ultimately accumulates in the Sargasso Sea waters. Similar results in the sampled area are also indicated by BGC‐Argo FDOM measurements which share similar optical properties with the matter accumulating in the ocean (Catalá et al., 2015; Zhao et al., 2017). Indeed, the fraction of measured FDOM to total CDOM (i.e., FDOM/K_bio_(380)) increases in summer when carbon biomass is the lowest and the abundance of picophytoplankton is the highest (see supporting information Figure S6). These results thus suggest that picophytoplankton may also act as a producer of CDOM that is potentially resistant to degradation, complement the microbial carbon pump (Jiao et al., 2010), and ultimately affect organic carbon sequestration.

## 4 Conclusions

High spatial‐ and temporal‐resolution data acquired for 6 years by BGC‐Argo floats have disclosed the main drivers of CDOM seasonal variability in the oligotrophic low‐chlorophyll NASTG. BGC‐Argo floats are therefore valuable observational tools to observe the ocean in a systematic way, which complement and enrich observations acquired from a variety of in situ and space‐based platforms (e.g., Iuculano et al., 2019; Morel et al., 2010) and modelling studies (Roshan & DeVries, 2017). In particular, the BGC‐Argo analysis based on bio‐optical and biogeochemical measurements have unveiled that:

- CDOM is mainly a direct product of phytoplankton metabolism;
- Winter mixing does not bring CDOM up from deep reservoirs;
- The impact of photodegradation is likely constant during all the year given the unlimited light;
- Picophytoplankton such as the genera *Prochlorococcus* and *Synechococcus* shapes CDOM temporal dynamics;
- Small phytoplankton will have a critical role under the ocean warming scenario. It will represent an increasing source of CDOM that, in turn, will impact on the ocean carbon cycle.

## Supporting information



Supporting Information S1
