# Supplementary material for: Small Phytoplankton Shapes Colored Dissolved Organic Matter Dynamics in the North Atlantic Subtropical Gyre
Source: Geophys Res Lett. 2019 Nov 8;46(21):12183–91. doi: 10.1029/2019GL084699 (PMC6916593; doi:10.1029/2019GL084699)
Supplement: Supplementary file 1 — Supporting Information S1 [file GRL-46-12183-s001.pdf]

Geophysical Research Letters

Supporting Information for

Small phytoplankton shapes colored dissolved organic matter dynamics in the North Atlantic subtropical gyre

Emanuele Organelli, and Hervé Claustre

Sorbonne Université, CNRS, Laboratoire d'Océanographie de Villefranche, LOV, F-06230 Villefranche-sur-Mer, France

Contents of this file

Figures S1 to S6
Tables S1 to S2

Introduction

This file contains supporting figures and tables. Figure S1 shows trajectories before quality-control of the four Biogeochemical-Argo floats operating in the North Atlantic subtropical gyre, between 2012 and 2018. Figures S2 to S6 show the monthly climatology of the non-water diffuse attenuation coefficients for downward irradiance (i.e., K_{bio}) and those for other hydrological, biogeochemical, optical and taxonomical variables within the mixed layer. Tables summarize statistics of analyses reported in the main manuscript. Methods are described in the main manuscript.

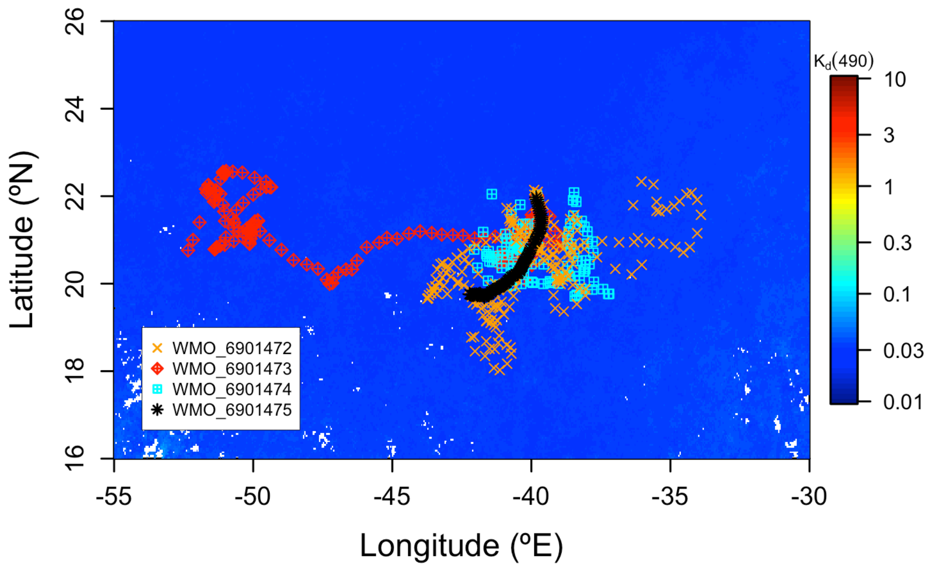

Figure S1. Trajectories of the four Biogeochemical–Argo floats operating in the North Atlantic subtropical gyre between 2012 and 2018, before quality control. Sampled stations (n=550) are superimposed onto the July 2014 Ocean Colour ESA Climate Change Initiative (v3.1) monthly composite of the diffuse attenuation coefficient for downward irradiance at 490 nm ($K_d(490)$; units of m^{-1}). Low $K_d(490)$ coefficients are associated with very clear waters.

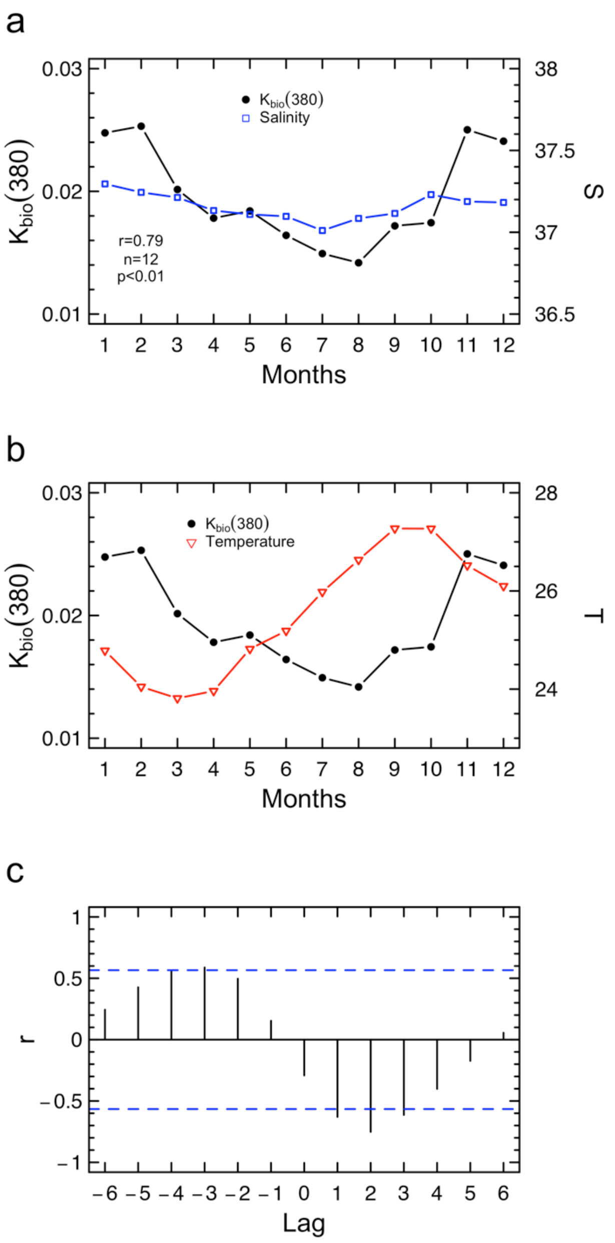

Figure S2. Monthly climatology within the mixed layer of the diffuse attenuation coefficient for downward irradiance at 380 nm ($K_{bio}(380)$; units of m^{-1}) and: **(a)** salinity (S; units of psu); and **(b)** temperature (T; units of ºC). In panel **(a)**, statistics for linear correlation between $K_{bio}(380)$ and salinity are shown. **(c)** Correlogram for cross-correlation analysis of $K_{bio}(380)$ *vs.* T. Dashed blue lines indicate the confidence level α=0.05. The cross-correlation analysis shows that $K_{bio}(380)$ is significantly negatively correlated with T, and causes changes in T with a 2 month lag (correlation coefficient r is equal to −0.75). The coefficients of variation (CV%) for each monthly average are listed in Table S1.

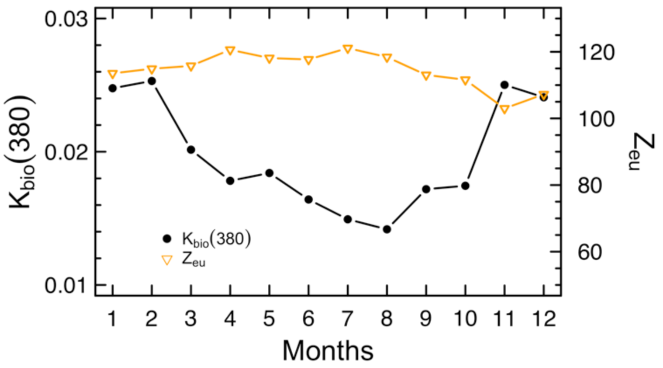

Figure S3. Monthly climatology within the mixed layer of the diffuse attenuation coefficient for downward irradiance at 380 nm ($K_{bio}(380)$; units of m^{-1}) and the depth of the euphotic layer (Z_{eu}, units of m). The coefficients of variation (CV%) for each monthly average are listed in Table S1.

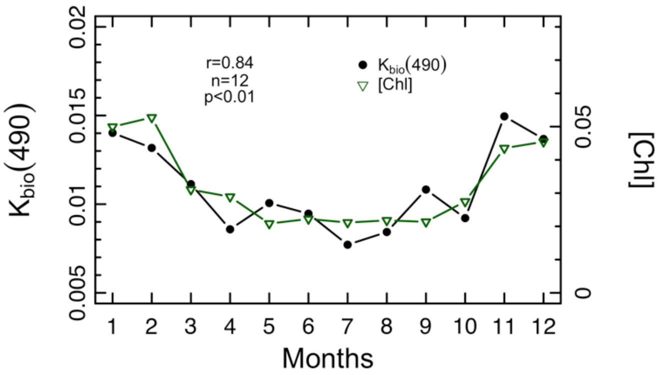

Figure S4. Monthly climatology within the mixed layer of the diffuse attenuation coefficient for downward irradiance at 490 nm ($K_{bio}(490)$; units of m^{-1}) and the concentration of phytoplankton chlorophyll as derived from fluorescence measurements ([Chl], units of mg m^{-3}). Statistics for linear correlation between $K_{bio}(490)$ and [Chl] are shown. The coefficients of variation (CV%) for each monthly average are listed in Table S1.

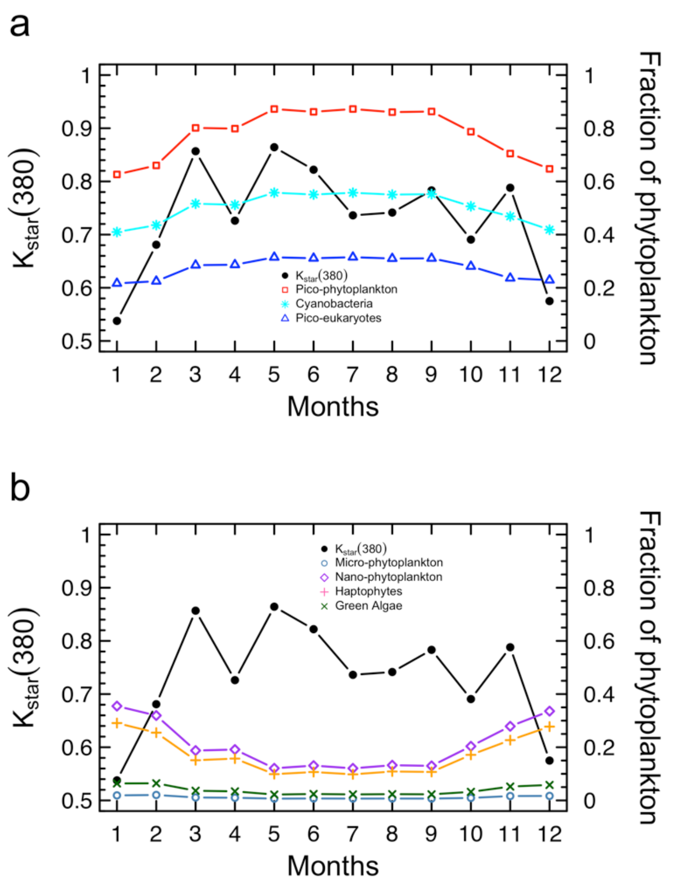

Figure S5. Monthly climatology within the mixed layer of the chlorophyll-specific diffuse attenuation coefficient for downward irradiance at 380 nm ($K_{star}(380)$; units of m^2 mg Chl^{-1}) and the relative fractions of **(a)** pico-phytoplankton, cyanobacteria and pico-eukaryotes; and **(b)** micro-phytoplankton, nano-phytoplankton, haptophytes and green algae. The coefficients of variation (CV%) for each monthly average are listed in Table S1.

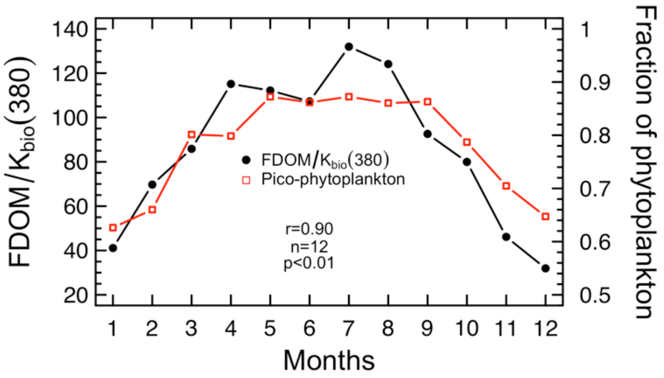

Figure S6. Monthly climatology within the mixed layer of the ratio between fluorescent dissolved organic matter measured with excitation/emission at 370/460 nm and the diffuse attenuation coefficient for downward irradiance at 380 nm (FDOM/K_{bio}(380); units of ppb of quinine sulfate/m^{-1}); and of the relative fraction of pico-phytoplankton. Statistics for linear correlation between FDOM/K_{bio}(380) and the fraction of pico-phytoplankton are shown. The coefficients of variation (CV%) for each monthly average are listed in Table S1.

CV%	Biogeochemical variables					Physical variables					
Month	K_{bio}(380)	K_{bio}(490)	[Chl]	K_{star}	FDOM/ K_{bio}(380)	T	S	MLD	Z_{NO3}	Z_{eu}	PAR_{ML}
1	20 (13)	14 (16)	32 (24)	45 (13)	50 (10)	2 (24)	0 (24)	33 (24)	9 (53)	7 (24)	37 (21)
2	14 (9)	31 (9)	59 (11)	56 (8)	9 (4)	2 (13)	0 (13)	52 (13)	11 (45)	4 (13)	32 (12)
3	23 (8)	36 (6)	66 (12)	35 (7)	31 (4)	2 (17)	0 (17)	58 (17)	13 (47)	8 (17)	39 (17)
4	18 (17)	28 (15)	48 (12)	38 (4)	3 (2)	2 (24)	1 (24)	54 (24)	10 (38)	4 (24)	38 (23)
5	25 (9)	32 (9)	24 (8)	24 (5)	– (1)	3 (19)	1 (19)	49 (19)	8 (38)	12 (19)	40 (18)
6	29 (14)	47 (15)	36 (8)	38 (6)	16 (3)	3 (19)	0 (19)	35 (19)	11 (39)	6 (19)	33 (18)
7	20 (13)	21 (10)	28 (17)	30 (12)	7 (2)	2 (21)	1 (21)	31 (21)	16 (34)	5 (22)	23 (19)
8	16 (10)	21 (12)	24 (11)	16 (4)	13 (4)	2 (21)	0 (21)	44 (21)	16 (30)	6 (21)	23 (19)
9	18 (11)	28 (10)	20 (13)	13 (9)	17 (3)	3 (18)	0 (18)	40 (18)	13 (22)	7 (18)	30 (16)
10	17 (20)	34 (19)	27 (28)	26 (19)	35 (9)	2 (30)	1 (30)	23 (30)	9 (55)	6 (30)	34 (30)
11	28 (7)	38 (8)	69 (11)	37 (6)	48 (4)	1 (13)	0 (13)	30 (13)	9 (43)	11 (13)	46 (11)
12	7 (11)	17 (11)	28 (13)	25 (9)	59 (6)	2 (15)	0 (15)	33 (15)	11 (50)	6 (15)	56 (15)

CV%	Taxonomical variables						
Month	Pico	Cyanobacteria	Pico-eukaryotes	Nano	Haptophytes	Green Algae	Micro
1	14 (24)	12 (24)	19 (24)	22 (24)	21 (24)	32 (24)	37 (24)
2	26 (11)	22 (11)	34 (11)	50 (11)	48 (11)	59 (11)	67 (11)
3	20 (12)	18 (12)	25 (12)	83 (12)	85 (12)	78 (12)	79 (12)
4	18 (12)	17 (12)	20 (12)	72 (12)	75 (12)	59 (12)	57 (12)
5	8 (8)	7 (8)	9 (8)	55 (8)	59 (8)	34 (8)	29 (8)
6	12 (8)	11 (8)	13 (8)	74 (8)	80 (8)	50 (8)	43 (8)
7	10 (17)	9 (17)	11 (17)	67 (17)	74 (17)	40 (17)	34 (17)
8	10 (11)	9 (11)	12 (11)	64 (11)	71 (11)	34 (11)	28 (11)
9	8 (13)	7 (13)	9 (13)	52 (13)	57 (13)	29 (13)	24 (13)
10	11 (28)	10 (28)	12 (28)	40 (28)	42 (28)	35 (28)	31 (28)
11	21 (11)	15 (11)	35 (11)	49 (11)	45 (11)	69 (11)	81 (11)
12	13 (13)	13 (13)	14 (13)	24 (13)	23 (13)	31 (13)	32 (13)

Table S1. Percent coefficients of variation (CV%) for each variable within the mixed layer, and month. For each month, CV% is the ratio between standard deviation and mean computed by using all quality-controlled samples collected by Biogeochemical-Argo floats between 2012 and 2018. In parenthesis, the number of samples used to compute each mean monthly value.

$K_{bio}(380)$ *vs.*	r-squared	Probability(>|t|)
[Chl]+MLD+PAR_{ML}+S	0.91	[Chl]**, MLD^{ns}, $PAR_{ML}{}^{ns}$, S^{ns}
[Chl]+MLD	0.90	[Chl]***, MLD^{ns}
[Chl]+PAR_{ML}	0.87	[Chl]***, $PAR_{ML}{}^{ns}$
[Chl]+Z_{NO_3}	0.87	[Chl]***, $Z_{NO_3}{}^{ns}$
[Chl]+S	0.87	[Chl]***, S^{ns}
MLD+PAR_{ML}	0.75	MLD^{ns}, $PAR_{ML}{}^{ns}$
[Chl]	0.87	[Chl]***
MLD	0.66	MLD***
PAR_{ML}	0.71	$PAR_{ML}{}^{***}$
Z_{NO_3}	0.25	$Z_{NO_3}{}^{ns}$
S	0.62	S***

Table S2. Statistics of multiple and simple linear regressions between the climatology of the diffuse attenuation coefficient for downward irradiance at 380 nm ($K_{bio}(380)$; units of m^{-1}) and biogeochemical and physical variables within the mixed layer. The r-squared of each regression and the significance of each variable (Probability(>|t|) are shown. ^{ns}: not significant; *: α=0.05; **: α=0.01; ***: α=0.001.
